# The influence of COVID-19 on agricultural economy and emergency mitigation measures in China: A text mining analysis

**DOI:** 10.1371/journal.pone.0241167

**Published:** 2020-10-23

**Authors:** Dan Pan, Jiaqing Yang, Guzhen Zhou, Fanbin Kong

**Affiliations:** 1 School of Economics, Jiangxi University of Finance and Economics, Jiangxi, China; 2 School of Economics and Management, Zhejiang A&F University, Zhejiang, China; Institute for Advanced Sustainability Studies, GERMANY

## Abstract

Understanding the influence of COVID-19 on China's agricultural economy and the Chinese government's emergency measures to ease the economic impacts of viral spread can offer urgently-needed lessons while this virus continues to spread across the globe. Thus, this study collected over 750,000 words upon the topic of COVID-19 and agriculture from the largest two media channels in China: WeChat and Sina Weibo, and employed web crawler technology and text mining method to explore the influence of COVID-19 on agricultural economy and mitigation measures in China. The results show that: (1) the impact of COVID-19 on China's agricultural economy at the very first phase is mainly reflected in eight aspects as crop production, agricultural products supply, livestock production, farmers' income and employment, economic crop development, agricultural products sales model, leisure agriculture development, and agricultural products trade. (2) The government's immediate countermeasures include resuming agricultural production and farmers' work, providing financial support, stabilizing agricultural production and products supply, promoting agricultural products sale, providing subsidies, providing agricultural technology guidance and field management, and providing assistance to poor farmers to reduce poverty. (3) The order of government's immediate countermeasures is not all in line with the order of impact aspects, which indicates that more-tailored policies should be implemented to mitigate the strikes of COVID-19 on China's agricultural economy in the future.

## 1. Introduction

As of May 8, 2020, Coronavirus Disease 2019 (COVID-19) has infected 3,938,064 people and killed 276,863 worldwide. It is exacerbating into the worst global public health emergencies. To control the rapid spread of COVID-19, cities and countries are gradually locked down, and citizens have been quarantined globally. Especially in China, a series of emergent control measures, such as city and population centers lockdowns, transportation control, closure of farmer markets, checkpoints, and roadblocks, have been adopted immediately throughout the country during the first two months [1]. These measures definitely have had a tremendous impact on China's agricultural economy. As one of the most critical sectors in the economy, agriculture can provide people with essential agricultural products to safeguard their livelihoods, which are the foundation of a stable society. Therefore, empirically investigating the impacts of COVID-19 on China's agricultural economy and exploring the Chinese government's emergency measures to ease these impacts are a prerequisite to the ongoing battle against COVID-19. It can not only assist policymakers in formulating effective policies but also can provide insights into the prevention efforts to similar infectious diseases in the future.

**However, to date, no study has empirically examined the impacts of COVID-19 on China**'**s agricultural economy or its** immediate **countermeasures. Only a few studies qualitatively explored the challenges China**'**s agricultural economy faced under the COVID-19.** For example, Jiang et al. [2] pointed out that COVID-19 has brought some adverse effects on the development of grain planting, livestock breeding, seed industry, leisure agriculture, agricultural products processing, vegetable industry, fruit industry, flower industry, and so on in China. They also proposed some countermeasures to reduce the negative impacts, such as ensuring the supply of grain and some to other essential agricultural products, encouraging the development of agricultural-socialized service organizations and online agricultural product selling, and strengthening financial support to the agricultural industry. Rozelle et al. [3] examined the economic and social impacts of COVID-19 on China's rural area based on 726 surveyed samples in seven provinces. They found that due to a radical decline in employment of rural farmers, rural households have lost around $100 billion in migrant worker wages and thus spend less on nutrition and health expenditures, which may become a potential challenge for their future development. Zhang et al. [4] pointed out that the COVID-19 has the potential to impact nearly every facet of the agricultural industry, including the massive unsalable agricultural products, the low development of livestock sector, the blocked supply of agricultural products, the obstruction of agricultural product trade, the decrease in farmers' income, and the increase in poverty in rural areas. Zhang et al. [5] analyzed the impact of COVID-19 on China's food supply and nutrition security. They pointed out that the hiccup in food and nutrition security is not severe in the short term because of the sufficient stocked food before the outbreak. However, the livestock industry and the processing enterprises have already been affected, and it may pose a grave threat to food supply and nutrition security for many people in the future. Yet, authors discussed potential measures include monitoring food prices and reinforcing market supervision, supporting the smooth operation of the logistical industry to guarantee food supply, providing financial supports to the agriculture industry to ensure the spring planting, protecting vulnerable groups—children, pregnant women, elderly people, and malnourished people, from getting worse. Zhu and Tian [6] investigated the impact of COVID-19 on China's grain production. They predicted that COVID-19 would not have a significant effect on grain production throughout the year, and the supply can be basically guaranteed. Still, due to the transportation blockages and labor shortage caused by the city lockdowns, the grain processing industry cannot restart production in the near future and is severely affected. Zhang [7] discussed the impact of COVID-19 on livestock farming in China. He stated that livestock farmers face severe pressure due to difficulties of the shortage of agricultural labor and raw materials, and the inadequate supply of animal feed. Rent reductions, financing support, and especially forced majeure certification are the preferred policies by livestock farmers to deal with the negative impacts of COVID-19. Shi et al. [8] analyzed the effects and countermeasures of the fisheries industry under COVID-19 from the aspects of production, circulation, consumption, and trade. They showed that COVID-19 has led to the rise prices of fishery raw material, the backlog of fresh aquatic products, the steep decline of aquatic product prices, and the shrink of aquatic products exports.

The literature above provided important understandings of the impacts of this novel virus on China's agricultural economy and the Chinese government's immediate mitigation measures. However, these studies mainly used traditional research methods, such as qualitative analysis or questionnaire surveys. Then, the results might be biased since the research questions were subjective and oriented and mainly depended on researchers. Moreover, due to the limited sample sizes, traditional research methods can only reflect the situation at a certain point, which cannot track the dynamic impacts of COVID-19 on China's agricultural economy. **Our research goes beyond these limitations in the literature. Based on over 750,000 words collected from WeChat and Sina Weibo, this study employed web crawler technology and text mining method to empirically estimate the impacts of COVID-19 on China**'**s agricultural economy and the Chinese government's** emergency **measures to address these impacts.**

**We contribute to emerging studies on the effects of COVID-19 in the following three ways**. First, no studies have ever used web crawler technology and text mining method to investigate the impacts of COVID-19 on China's agricultural economy and the government's emergent mitigation measures. We fill this gap and contribute to future studies. The conclusions drawn from these methods are more objective and can provide more valuable and applicable suggestions for policymakers [9]. Also, the web crawler technology and text mining method used in this study might be applied to other similar epidemics or in other countries. Second, this study investigated the impacts of COVID-19 on China's agricultural economy and the corresponding emergency countermeasures from nearly every facet of the agricultural industry, which can comprehensively reveal the impacts of COVID-19 on China's agricultural economy. Third, given that COVID-19 continues to spread worldwide, the results of this study can provide substantial implications for other countries that have taken similar measures.

The rest of this paper is structured as follows. Section 2 briefly describes the data and method. Section 3 presents the empirical results and offers a discussion. Section 4 proposed some policy implications based on the results from Section 3. Section 5 outlines the conclusions and future research directions.

## 2. Data and method

The original data are derived from WeChat (https://weixin.sogou.com/) and Sina Weibo (https://s.weibo.com/). And we state that the collection method complied with the terms and conditions for the website.

### 2.1. Data collection

We retrieved online news until the end of March (including news reports, public opinion, official documents, and academic review and analysis) about the impact of COVID-19 on China's agricultural economy from two major social media platforms: WeChat and Sina Weibo. The main reasons we chose WeChat and Sina Weibo as our data source are as follows. First, these two social media platforms have the highest usage rate among Chinese Internet users. It was reported that the monthly active users for WeChat were 990 million and 465 million for Sina Weibo in 2019, which accounts for about 71% and 33% of China's total population, respectively. Therefore, most people could have received news of COVID-19 mainly from WeChat and Sina Weibo. Second, almost all major media in China have their own WeChat and Sina Weibo official accounts so that we could get access to nearly all reports about COVID-19 through these two.

Specially, we employed web crawler technology to collect targeted information. Web crawler technology is an efficient and convenient technology that can automatically obtain needed information from a large number of web pages, which is now widely used in various research fields [10, 11]. The processes of web crawler technology in our paper are as follows:

1. Imitated login. We used the username and password to access to WeChat and Sina Weibo.
2. Select news according to a specific strategy. We then chose the topic keywords of "COVID-19", "agriculture", "rural areas", and "farmers".
3. Store the selected news. Necessary information on each selected news, such as the news title, news text, the published time, and the publisher, are stored.
4. Export stored information. After collection, we then clean the data to eliminate duplicated news and delete irrelevant news. Then all the information was exported and coded.

We crawled online news during the first two months from January 25, 2020, to April 1, 2020. In total, we get 337 WeChat articles and 490 Weibo articles with a total number of approximately 750,000 words.

### 2.2. Text mining method

**Fig 1:**
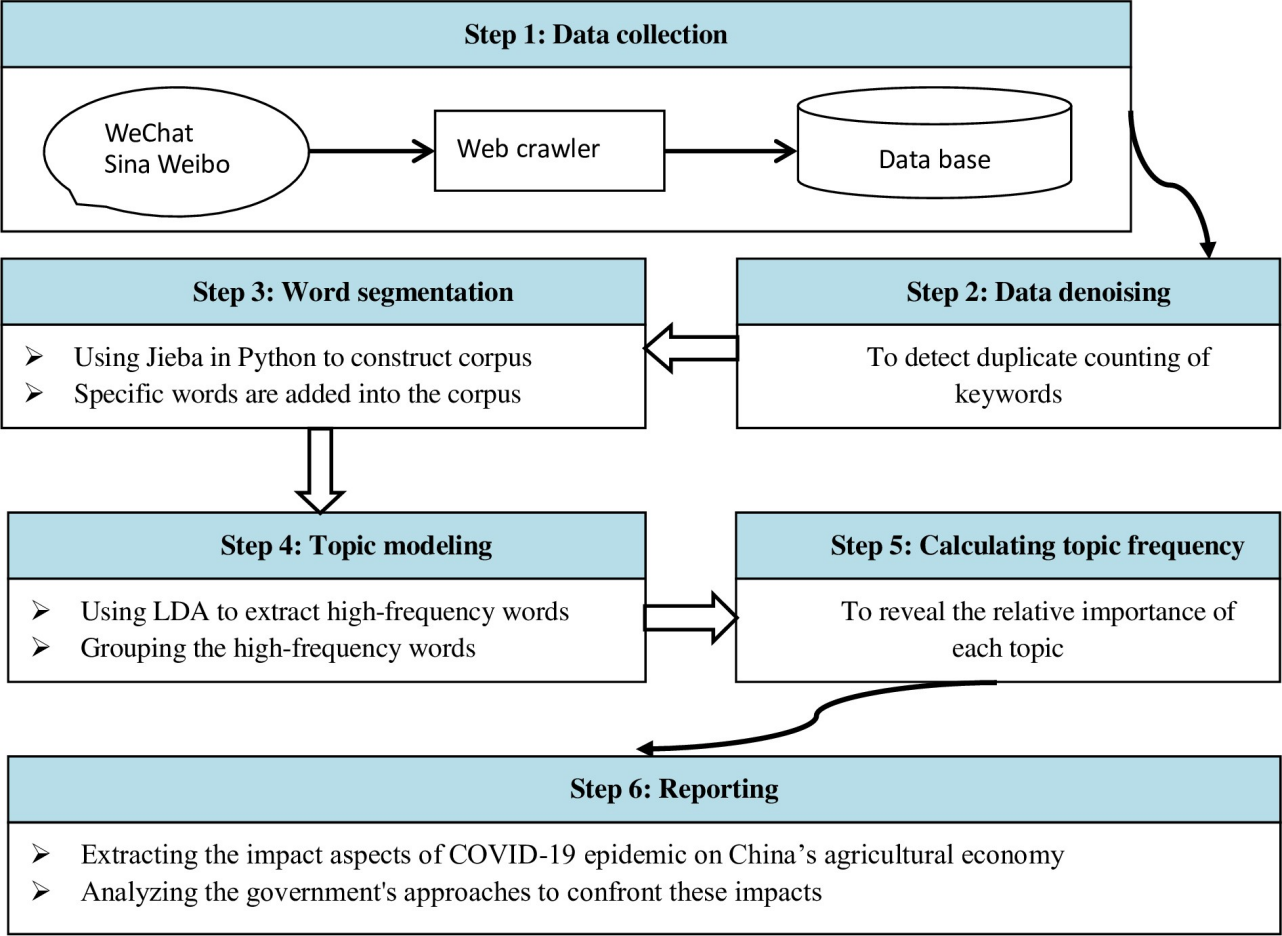
The processes of text mining method.

Text mining method is a popular text analytical technique aiming to extract knowledge and relationships from a large number of textual documents [12, 13]. Recently, this method was applied for the resource optimization in agriculture and crop [14–17]; however, it has not been utilized to understand the influence of COVID-19 on agricultural economy and mitigation measures in China. The processes of text mining in our paper are shown in Fig 1.

1. Data denoising. Denoising was used to avoid duplicate counting of keywords. In this study, we first combine the keywords with the same meaning, e.g., "Corona Virus Disease 2019" and "COVID-19", "farming", and "agricultural production"; and then we delete the punctuation marks and spaces in the text, leaving only the text content for analysis.
2. Word segmentation. Word segmentation is used to build a corpus that can reflect the aspects of the impact of COVID-19 and the government's responses to the effects. Specifically, we adopt the word segmentation tool of Jieba based on Python to construct the corpus. Some new words, such as "COVID-19", "rice bag", and "vegetable basket" were added into the corpus to improve the accuracy of word segmentation.
3. Topic modeling. We used the Latent Dirichlet allocation (LDA) method—one of the most popular topic modeling technologies, to discover the main topics from the substantial text content. LDA assumes that a document is comprised of a mixture of words regarding potential topics, and each topic can be considered as a multinomial distribution of words [18]. In this study, first, LDA was used to perform word frequency statistics on the text to obtain meaningful high-frequency words, and then we grouped the high-frequency words based on their meaning and distribution. Finally, we named each group according to the high-frequency words in the group.
4. Calculating the topic frequency. Based on the topic generated, we then calculated the frequency of each topic to reveal the relative importance of each topic. The function is as follows: *F_i_* = *d_i_/D*, where *d_i_* is the number of high-frequency word *t_i_* in the text analyzed, *D* is the total number of all high-frequency words in the text analyzed.
5. Reporting results. We report the information about the impact aspects of COVID-19 on China's agricultural economy and the government's approaches to confronting these impacts that are retrieved from the analysis above.

## 3. Results and discussion

### 3.1. The statistical description of the post number of COVID-19 on China's agricultural economy

**Fig 2:**
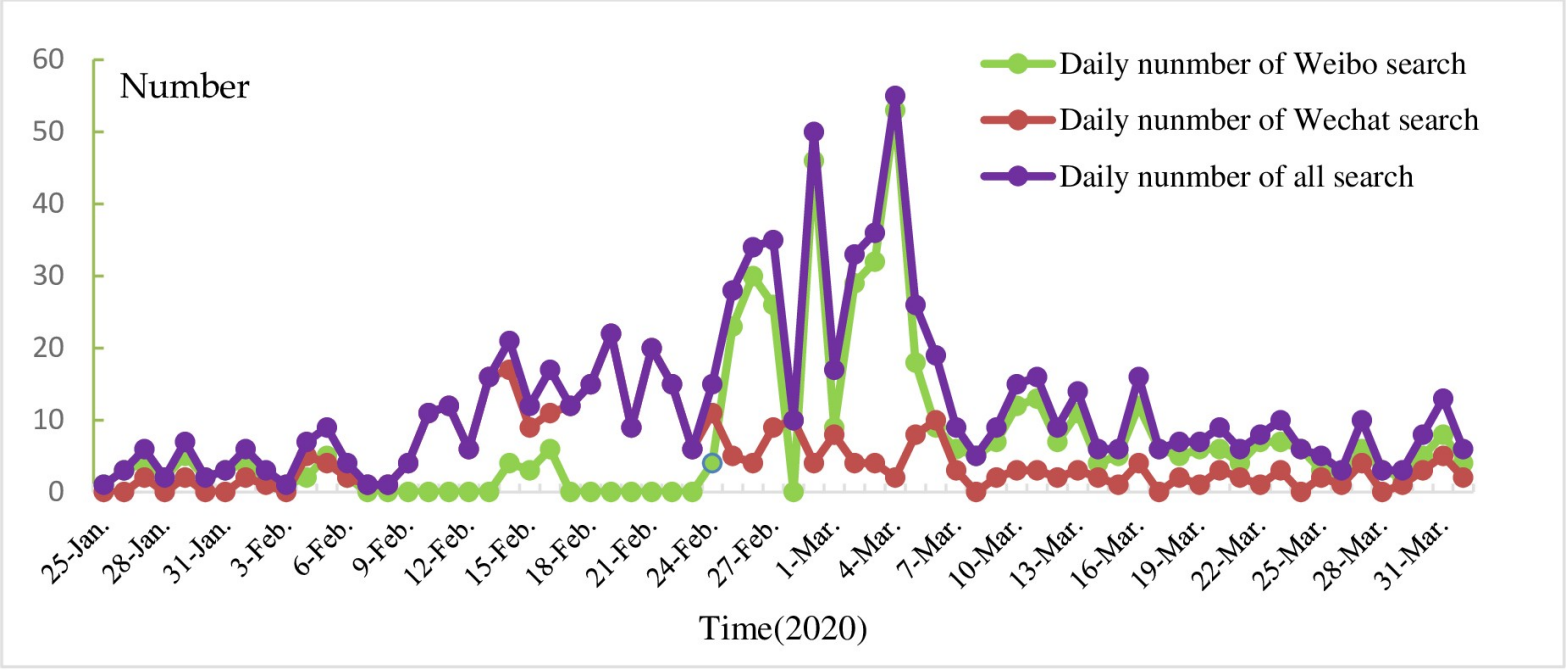
The post number of the impact of COVID-19 on agriculture development.

Before the text mining analysis, we collected and analyzed the post numbers of reports upon the topic of the society's concern and its trend regarding impacts of COVID-19 on the agricultural economy, as presented in Fig 2. It showed that before February 13, the total number of media articles was relatively few, only 89, and the number of daily articles was only 4.7. After February 13, the number of articles gradually increased and peaked on March 4. The reason for this trend is that an official policy "*Notice on the Mayor Responsible System for Compacting Vegetable Baskets and Stabling Agricultural Products Supply*" was issued by the government on February 12, 2020, which makes the impact of COVID-19 on agricultural development attracted more attention from the mass media.

### 3.2. The impact aspects of COVID-19 on China's agricultural economy

**Fig 3:**
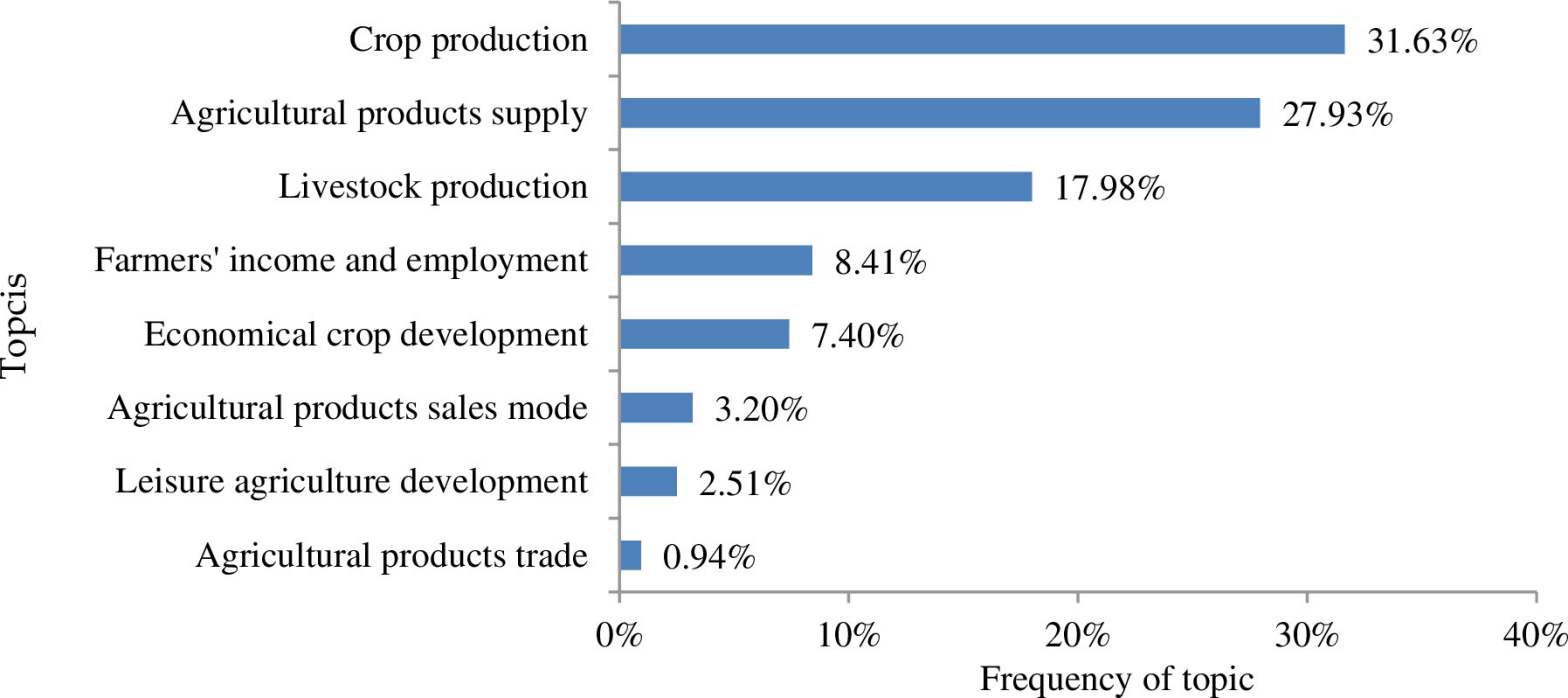
Topic frequency of impacts of COVID-19 on China's agriculture economy.

Based on the high-frequency words retrieved from the topic modeling resulted from the text-mining process, the impacts of COVID-19 on China's agricultural economy can be summarized into eight aspects. According to the topic frequency, the order of these eight aspects followed the sequence of crop production (31.63%), agricultural products supply (27.93%), livestock production (17.98%), farmers' income and employment (8.41%), economic crop development (7.40%), agricultural products sales model (3.20%), leisure agriculture development (2.51%), and agricultural products trade (0.94%). The high-frequency words in each topic and their counts are shown in Table 1, and the frequency of each topic is shown in Fig 3. The counts of high-frequency words in the topic of crop production is the highest (5151), with a topic frequency of 31.63%, accounting for almost one-third of the total frequency in all eight topics; agricultural products supply ranks second (4549), with a topic frequency of 27.93%; livestock production ranks third (2929), with a topic frequency of 17.98%. These three topics are the main aspects reflecting the impacts of COVID-19 on the agricultural economy, which accounts for 77.55% of total topic frequencies. In the rest of this section, we will analyze the impacts of COVID-19 on the agriculture economy by each topic in detail.

**Table 1:** Topics about the impacts of COVID-19 on China's agriculture economy.

Topics	High-frequency words
Crop production	Spring plowing preparation (1776), crop production materials (902), agricultural production (858), fertilizer (357), seed and seedling (288), food (263), pesticides (236), wheat (133), rice (114), corn (68), rape (62), agricultural labor (94)
Agricultural products supply	Agricultural products (1340), agricultural products supply (987), agricultural products transportation (793), agricultural products sales (587), vegetable baskets (350), agricultural products prices (326), slow sales (135), rice bags (31)
Livestock production	Breeding (611), livestock (545), live pigs (361), aquatic products (327), feed (311), poultry (308), slaughter (250), wildlife farming (132), dairy industry (84), eggs (49), pork (49)
Farmers' income and employment	Poverty alleviation (256), poverty (208), income increase (149), farmers' income (37), migrant workers (186), migrant workers (126), start of work (120), employment (191), return to work (97)
Economical crop development	Vegetables (1009), fruits (141), strawberries (55)
Agricultural products sales model	E-commerce (140), online sales (112), internet (140), direct supply (42), orders (87)
Leisure agriculture development	Leisure agriculture (235), rural tourism (110), products picking (63)
Agricultural products trade	Export (50), processing enterprise (64), trade (39)

Note: The figures in brackets are counts of high-frequency words.

#### 3.2.1. Crop production

COVID-19 has the most significant impact on crop production so far according to the result, mainly reflected in the following four aspects with 12 high-frequency words:

① **Spring plowing preparation is the basis of ensuring sufficient food supply and is the priority work in spring whenever the virus is spreading and now is in worry**. Related high-frequency words include spring plowing preparation (1776), and agricultural production (858), accounting for 51.14% of the total frequency in crop production. With the arrival of spring, spring plowing preparation will gradually start from south to north. Because of seasonality, farmers must start spring plowing as soon as possible to avoid agricultural income loss. This is not only hurting the welfare of the 40% Chinese people whose revenue are mostly relying on agriculture, but also affecting the stability of food supply. According to estimates by experts from the Rice Research Institute of the Chinese Academy of Agricultural Sciences, if the spring sowing date of rice in southern China is delayed by COVID-19 in 2020, the rice production in this region will be reduced by at least about 5% [2].
② **Crop production materials include seeds, fertilizers, pesticides, and other agricultural inputs within the prescribed time, and it is now difficult due to continuous control measures for COVID-19—such as prohibiting companies from resuming work and closing roads since late January**. Related high-frequency words include agricultural inputs (902), fertilizer (357), seed and seedling (288), and pesticides (236). According to a survey by China Seed Association, nearly 40% of seed enterprises have not sold any seeds after the outbreak. Meanwhile, the crop production materials are difficult to enter villages due to the city lockdowns. It is reported that only 3.3% of seed stores are in regular operation since early February this year. Therefore, the shortage of crop production materials has become one of the main obstacles in agricultural production during the outbreak of this new coronavirus.
③ **Grain crop production of the three major grain crops—rice, wheat, and corn have also been hindered by COVID-19 due to obstruction of spring plowing and insufficient supply of crop production materials.** Related high-frequency words include food (263), wheat (133), rice (114), corn (68), and rape (62). At the same time, spring is also a crucial moment for rape cultivation. Thus the breeding and production of rapeseed under the influence of COVID-19 has also received some attention.
④ **Agricultural labor is in short due to quarantine and closure across the country after the outbreak of COVID-19**. A related high-frequency word is agricultural labor (94). It is known that China's agricultural production relies heavily on the manual labor force. Therefore, this agricultural labor shortage then exposed the shortcomings of this low-automation production model. Restricted by the movement of people, many agricultural enterprises require a lot of labor during spring plowing, but it is difficult for labors coming back to work or for enterprises to hire new workers, which poses a significant challenge for agricultural production. For example, a survey asking about agricultural production preparation in Quzhou City by the National Bureau of Statistics found that only 61.5% of hired workers can come to work during the COVID-19 period.

#### 3.2.2. Agricultural products supply

Eight high-frequency words are highly related to agricultural products supply: agricultural products (1340), agricultural products supply (987), agricultural products transportation (793), agricultural products sales (587), vegetable baskets (350), agricultural products prices (326), slow sales (135), and rice bags (31). It accounts for 27.93% of the total topic frequencies amongst all topics, as shown in Fig 3.

The unprecedented isolation measures taken to curb the spread of COVID-19 have hurdled most of the country's food supply chain. The specific performance is as follows: on the supply side, due to the strict traffic control implemented in various areas, products cannot go out, raw materials cannot enter, transportation of agricultural products is not smooth, and then the supply is reduced; on the demand side, households now with uncertain incomes and restricted access to supermarkets will reshape their demand. Consumers are reported to likely to change their dietary patterns by disproportionately large consumption declines, that is, to reduce the demand for fruits, vegetables, and other perishable foods and only buy those agricultural products that are necessary to maintain their basic needs during the particular period. The overall market demand for agricultural products has declined compared to regular seasons.

These changes in food supply and demand have led to the importance of a sufficient supply of basic agricultural products. Two emerging high-frequency words—vegetable baskets and rice bags—have been put forward by the Chinese government. Among them, the frequency of vegetable baskets is 350, which is much higher than the frequency of rice bags (31 times), indicating that the supply of agricultural products in "vegetable baskets" is more concerned by people than the supply of agricultural products in "rice bags".

#### 3.2.3. Livestock production

There are eleven high-frequency words related to livestock production: breeding (611), livestock (545), live pigs (361), aquatic products (327), feed (311), poultry (259), slaughter (250), wildlife farming (132), dairy industry (84), eggs (49), and pork (49), accounting for 18.29% of the total topic frequencies amongst all topics.

The livestock industry has the characteristics of a short life cycle, long industrial chain, and large feeding volume. Due to considerable measures are taken to control the COVID-19, such as blockading villages, delaying the resumption of feed mill operations, suspending trading, and closing down slaughterhouses, the livestock industry has been seriously upended, and the livestock farmers have suffered significant economic losses.

Specific performance can be reflected in the following four facets:

① **Feed supply is facing shortages in the livestock industry because of strict traffic control and the suspension of feed production.** Related high-frequency word is feed (311), breeding (611), and livestock (545). Farmers need to purchase feed to meet the daily dietary requirements of livestock and poultry, and it is presently difficult due to closedowns of feed enterprises. According to a survey from the Enterprise Survey for Innovation and Entrepreneurship in China on some enterprises in the agricultural sector, 38.5% of livestock farmers list feed shortages resulting from "logistics disruption" as the biggest challenge in their production after COVID-19 breakout. Feed shortages also indicate that animals and poultry may starve to death. It is reported that millions of chickens in Hubei province have been wiped out due to the insufficient supply of feed, with some farmers had to euthanize young birds, while others had halved the amount of feed fed per bird. It would be deathly damage to farmers' economic revenues.
② **Livestock slaughter trading and markets have been shuttered in many regions since livestock is considered to be a potential carrier of COVID-19.** Related high-frequency words are slaughter (250), eggs (49), and pork (49). According to the official statistics, all or some cities and counties in 27 provinces across the nation closed the livestock and poultry trading and slaughter markets during the peak of COVID-19 in China. In addition to the shutdown of livestock and poultry slaughtering enterprises, the limited movement of people also causes labor shortage in some local slaughterhouses and thus makes it difficult for livestock slaughtering activities to be carried out regularly.
③ **Livestock production of all types was affected, and related topic frequency order followed by the sequence of live pigs (361) > aquatic products (327) > poultry (308) > dairy industry (84).**

First, for live pig production, the impact of the 2019 outbreak of African swine fever on the live pig industry has not subsided, and COVID-19 has caused the live pig industry to be hit again. COVID-19 has hindered the transfer of pig feed in some areas, resulting in an insufficient supply of pig feed. Most of the newly constructed pig farms are in a state of shutdown, and farmers' enthusiasm for pig replenishment decreases. The difficulty of breeding pigs or piglets replenishment is also significantly increased.

Second, for aquatic products, due to transport control, closure of farmers' markets, and marketing docking, some aquatic products are seriously unsalable. Among them, aquatic products with large breeding volume and short production cycles, such as tilapia, prawns, crayfish, and bulk freshwater fish, are particularly affected.

Third, for the poultry industry, the impact of COVID-19 is especially large. The reasons may be as follows: on the one hand, the rising pig prices in 2019 have caused many poultry farming enterprises to expand production significantly, and thus the production capacity of the poultry industry has reached a high level before the outbreak of COVID-19. On the other hand, compared with other livestock industries, the production cycle of poultry farming is shorter, and the feeding volume is higher. During the COVID-19 period, traffic restrictions have hindered the transportation of feed and live poultry, and the closure of live poultry trading markets has prevented poultry from being sold. Therefore, the phenomenon of the live burial of chicken seedlings and stuffed sheds of chickens and ducks has appeared in many places, which causes great losses to farmers.

Fourth, for the dairy industry, most farmers are facing multiple challenges such as declining demand for dairy products, shortage of feed reserves and supply, and labor shortages, which have led to the phenomenon of "pouring milk" in some regions to prevent the sale of raw milk at low prices.

④ **Wildlife farming has been much negatively and directly affected by COVID-19.** A related high-frequency word is wildlife farming (132). The breeding of wildlife is psychologically rejected by consumers because wildlife is suspected to be one of the primary sources of COVID-19. The National Forestry and Grassland Administration issued "*Emergency Circular on Further Strengthening Wildlife Control*" on January 30, 2020, which stipulates that all administrative approvals for the transportation, sale, operation, and utilization of wildlife should be closed until COVID-19 has been controlled. Therefore, many provinces and cities have explicitly banned the transshipment and trafficking of wildlife in artificial breeding sites and all forms of wildlife trade during the COVID-19 period. The public also strongly demanded that the country should introduce laws to prohibit and control wildlife trade after COVID-19.

#### 3.2.4. Farmers' employment and income

There were nine high-frequency words pertaining to farmers' employment and income: poverty alleviation (256), poverty (208), income increase (149), farmers' income (37), migrant workers (186), work (126), start working (120), employment (191), and return to work (97), accounting for 8.41% of the total frequencies amongst all topics.

COVID-19 triggered industrial fluctuations that spread to farmers' job market and affected their incomes. This is manifested in the following three aspects:

① **Migrant workers' employment has confronted severe impact due to disruption of return transportation and restrictions in labor-importing areas on the entry of migrant populations during the period of COVID-19.** In the short term, farmers are unable to go out for work. Related high-frequency words include migrant workers (186), work (126), start working (120), employment (191), and return to work (97). Especially, provinces with a high number of migrant workers, such as Hubei, Henan, Chongqing, Sichuan, Hunan, Jiangxi, Anhui, and Chongqing, are particularly affected. Meanwhile, the main employment areas of these migrant workers are in catering, construction, and service industries, which are seriously influenced by COVID-19. Data from the 2019 Statistical Bulletin of National Economic and Social Development show that the total number of migrant workers in China in 2019 was 290 million, and almost all of these migrant workers cannot go to work because of COVID-19.
② **Farmers' income had severely affected by COVID-19 on two major components: wage income and agricultural income.** Related high-frequency words are income increase (149) and farmers' income (37). Firstly, COVID-19 has reduced the wage income of farmers by affecting their migratory work. In the urban area, some white-collar workers can still get salaries if working from home during the quarantine. However, most rural workers are blue-collar and cannot work from home, and then they cannot get paid. It is reported that almost every person in rural areas had stopped working—either working in a city as a migrant laborer or in their local counties as an off-farm worker [3]. The closure of workplaces and the transportation restrictions force farmers to stop working. Secondly, the difficulty in finding housing and fear of second quarantine in workplaces also hindered migrant laborers from working. The sharp decline in employment decrease migrated farmers' income since that in the urban area of China, workers can still get a salary if they do not work during the quarantine as required by China's state council; however, most rural workers cannot get paid because of their meager social safety net. It is estimated that farmers will lose around $100 billion in wages after one month of quarantine, which is more than the total global economic loss of SARS [3]. Thirdly, due to quarantine and transportation restrictions, farmers' local agricultural activities cannot continue, and the agricultural products cannot be sold, which indicates a radical decline in agricultural income for the entire year.
③ **Poverty alleviation is a primary goal of China in 2020, and however, the radical decrease of farmers' income makes it more challenging to achieve this goal.** Related high-frequency words are poverty alleviation (256) and poverty (208). First, farmers are hindered from going out to work. Poor peasant families whose primary income source coming from the migrant work may be harder to decrease poverty. Second, the development of pro-poor industries is blocked, which may also affect the progress of poverty alleviation for poor households. The industries that play a crucial role in poverty alleviation, such as livestock production, wildlife farming, seasonal agricultural products planting, have suffered a great impact from COVID-19, affecting poor farmers' incomes.

#### 3.2.5. Economic crop development

Three high-frequency words were related to economic crop development: vegetables (1009), fruit (141), and strawberries (55), accounting for 7.4% of the total frequencies amongst all topics. Economic crops such as vegetables and fruits are typical fresh products with strict shelf life, suffered a severe impact by the COVID-19.

① **Vegetable crop as an essential agricultural product for citizens is affected by COVID-19.** A related high-frequency word is vegetables (1009). First, due to shortage of agricultural production materials—such as seed, fertilizer, pesticides—and restrictions of transportation, ongoing production of vegetables becomes impossible, and a large number of vegetables that are ripe and about to ripen could not be sold normally, and some even rotted in the fields; Second, cessation of most catering and vegetable processing enterprises led to a sharp decline in vegetable demand and a severe product backlog. During the COVID-19, vegetable prices are required to remain stable under the regulation of government. However, in the long run, the shortage of workers will increase the production costs of the vegetable industry and the withdrawal of some producers, which may cause a potential risk of a generous increase in vegetable prices.
② **Fruit crop marked by its highly seasonal nature and the associated need for adequate storage and distribution logistics was therefore greatly affected by COVID-19 because of transportation restrictions.** Related high-frequency words are fruits (141) and strawberries (55). It is difficult for fruit farmers to transport the fruit, because the quarantine has caused many truck drivers being trapped at home. Some special seasonal fruit industries, such as citrus and strawberries, were particularly affected by COVID-19. For example, the Spring Festival period is the peak period of strawberry picking, but now has led to a decline in sales of picking, and then farmers' incomes have fallen sharply. More seriously, a large proportion of small and medium-sized farmers suffered heavy losses due to weak storage and product processing capacity. It is reported that the price of strawberry fell by 48% compared with that time before COVID-19 and fell by 50.2% compared with the same period of last year.

#### 3.2.6. Agricultural products sales model

There were five high-frequency words in the topic of agricultural product sales model: e-commerce (140), online sales (112), internet (140), direct supply (42), and orders (87), accounting for 3.20% of the total frequencies amongst all topics.

The agricultural products sales model has been updated due to COVID-19. Before the COVID-19 outbreak, according to the Chinese Industry Information Report, farmers' markets and supermarkets accounted for 93.8% of Chinese consumers' fresh food sources in 2018, while fresh food e-commerce accounted for only 4.9%. During the COVID-19 outbreak, people worried about cross-infection and were reluctant to go to public places to buy food, which stimulates the development of e-commerce platforms, such as "*Freshippo*", "Ding-dong to buy vegetables", "Missfresh", and other platforms. According to the survey, Missfresh's transaction volume increased by 321% from January 24 to January 28. In cities such as Guangzhou, Shenzhen, and Chengdu, Freshippo's orders were 5 to 10 times more than usual. In the long run, if e-commerce platforms continue to improve their warehousing and distribution capabilities, Chinese consumers, especially young people, will continue to purchase food online, and online sales will gradually become Chinese people's consumption habits.

#### 3.2.7. Leisure agriculture development

There were three high-frequency words related to leisure agriculture development: leisure agriculture (235), rural tourism (110), and products picking (63), accounting for 2.51% of the total frequencies amongst all topics.

Leisure agriculture is an industry that combines agricultural production and leisure recreation. Similar to tourism, leisure agriculture is based on interaction amongst tourists, which is greatly affected by COVID-19. Specifically, to restraint the spread of COVID-19, the government implements closures of all eco-parks, scenic spots, bed and breakfasts, and leisure agricultural activities such as eco-catering, sightseeing experience, picking fruit, and fishing, which caused a massive loss in leisure agricultural enterprises. It is reported that the income of all 156 leisure agricultural enterprises in Guangzhou province during the COVID-19 period is zero, while this number was $0.33 billion in 2018 [2].

#### 3.2.8. Agricultural products trade

Three high-frequency words were related to agricultural products trade: export (50), processing enterprises (64), and trade (39), accounting for 0.94% amongst all topics. The impacts of the pandemic on China's agricultural products trade, especially export, are mainly through the following two aspects:

First, the strict border control of China's main export countries has severely restricted the export of agricultural products. Although the World Health Organization has proposed that after the outbreak, "there is no reason to take unnecessary measures to interfere in international travel and trade", many countries have nevertheless adopted restrictive measures. As of April 8, 2020, a total of 35 countries (regions) have taken control measures on China's agricultural products trade. For example, Nepal suspended the import of fruits from China; Turkey and the Philippines temporarily prohibited the import of poultry products from China; Mongolia suspended the import of chicken and egg products from China and strengthened the quarantine supervision of imported goods from China; India has also stepped up quarantine restriction on agricultural products and livestock products from China, requiring all relevant products are sent to the laboratory for testing; the United States, Russia, and other countries have also strengthened their supervision of China's agricultural products. Besides, a series of restrictive measures in various countries have prolonged the transportation and customs clearance time for exported China's agricultural products, resulting in an increased risk of default on export contracts. Due to the short shelf life and freshness of agricultural products, these strict regulatory measures have significantly increased the economic losses of exported products. China Rural Network reported that 72.8% of the interviewed agricultural enterprises faced the cancellation of signed orders, while 14.1% of them have over 50% cancellation rate of signed orders [19].

Second, the interruption of the domestic agricultural product supply chain is also one of the key factors hampering exports. During the epidemic, supply chain of exported agricultural products was blocked due to delays in the resumption of work and restrictions on the movement of personnel. Nowadays, with the reversal of the domestic epidemic prevention, the supply chain for agricultural exports has now recovered by more than 80%, but it still faces delays in export delivery progress.

As a result, agricultural products trade has declined significantly after the COVID-19 outbreak. According to data from the General Administration of Customs of China, the total value of agricultural exports was $9.488 billion from January to February 2020, a decrease of 11.58% compared with a year ago. The total value of agricultural exports and imports was $34.147 billion, a sharp decline of 23.11% compared with that in November-December 2019.

### 3.3. Chinese government's emergency countermeasures to ease the impacts of COVID-19

**Fig 4:**
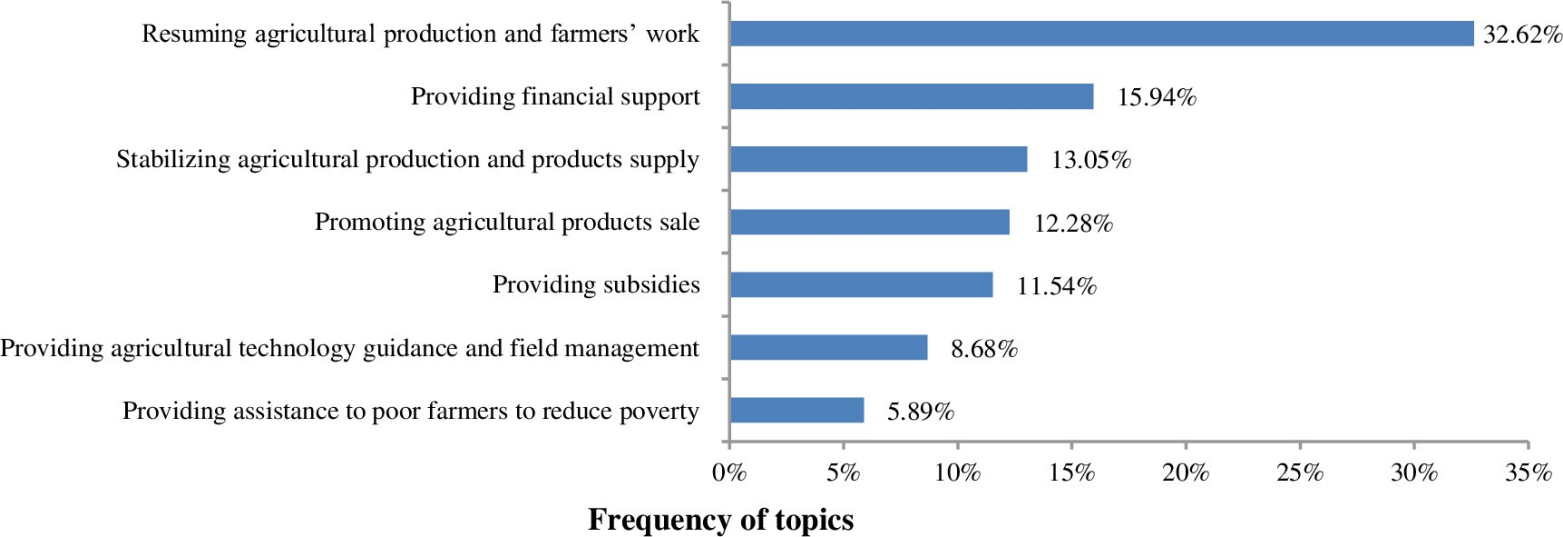
Topic frequency of Chinese government's countermeasures to ease the impacts of COVID-19.

Based on the high-frequency words retrieved from topic modeling resulted from the text-mining process, the Chinese government's approaches to ease the impacts of COVID-19 during the first two months can be summarized into seven aspects. According to the topic frequency, the order of these seven aspects followed the sequence of resuming agricultural production and farmers' work (1312), providing financial support (641), stabilizing agricultural production and products supply (525), promoting agricultural products sale (494), providing subsidies (464), providing agricultural technology guidance and field management (349), providing assistance to poor farmers to reduce poverty (237). The high-frequency words in each topic and their counts are shown in Table 2, and the frequency of each topic is shown in Fig 4. We also outlined the representative policies corresponding to each topic in S1 Table. In the rest of this section, we will analyze the government's countermeasures to mitigate the impacts of COVID-19 by each topic in detail.

**Table 2:** Topic Classification of government emergency measures to address the impacts of COVID-19.

Topic category	High-frequency words
Resuming agricultural production and farmers' work	Resuming farmers' work (831), recovery agricultural production (481)
Providing financial support	Loan (245), finance (144), discounted interest (104), financing (76), credit (72)
Stabilizing agricultural production and products supply	Ensure supply (347), stable production (178)
Promoting agricultural products sale	Production and sales docking (170), allocation and transportation (130), green channel (119), point-to-point (75)
Providing subsidies	Subsidy (246), allowance (170), guaranteed purchase (48)
Providing agricultural technology guidance and field management	Agricultural technology (128), pest management (106), field management (66), plant protection (49)
Providing assistance to poor farmers to reduce poverty	Poverty alleviation (162), assistance and support (75)

Note: The figures in brackets are the counts of high-frequency words.

#### 3.3.1. Resuming agricultural production and farmers' work

Resuming agricultural production and farmers' work is primary at present. Two high-frequency words are highly related to this topic: resuming farmers' work (831) and recovery agricultural production (481), with a topic frequency of 32.62%, accounting for almost one-third of the total topics. Specifically, this topic is mainly reflected as follows:

① **Help agricultural enterprises to resume production**. Under the premise of epidemic prevention, the Chinese government had given priority to speed up the production resumption of major agricultural enterprises, such as agricultural materials production enterprises, agricultural products processing, and distribution enterprises, slaughtering enterprises, new-type agricultural operators, and other production enterprises.
② **Encourage farmers to work in local areas**. The government has encouraged local businesses, cooperatives, and family farms to employ local laborers. Some provinces have specifically issued policies to encourage migrant workers to work in local areas. For example, Hunan Province implemented several approaches—such as absorbing employment, enhancing farmers' ability, living and transportation subsidy, supporting entrepreneurship, developing temporary jobs, guaranteeing social rights, and promoting farmers' employment in local areas.
③ **Serve migrate workers directly**. A "point-to-point" service of directly transporting migrant workers to the workplaces by using chartered buses has played a key role in promoting the employment of migrant workers. This "point-to-point" service has been implemented in 27 provinces in China. Through direct transportation, the whole process can be traced, which significantly increases the efficiency of work resumption during the COVID-19. It is estimated that a total of 2.63 million migrant workers has been transported by "point-to-point" service all over the country until March 6, 2020.

#### 3.3.2. Providing financial support

Providing financial support is one of the central policies to help enterprises effectively cope with the impact of COVID-19. There are five high-frequency words related to this topic: loan (245), finance (144), discounted interest (104), financing (76), and credit (72), accounting for 15.94% of the total frequencies amongst all topics. The specific measures of financial support are as follows:

① **Provide a loan discount**. Providing temporary loan discount support policies, in terms of customer access, loan term, loan amount, and guarantee mode, to the following enterprises—such as absorbing large numbers of migrant workers that are greatly affected by COVID-19 and have temporary business difficulties, to ensure that they are can get a loan quickly.
② **Provide credit support**. Specific measures include: implementing favorable policies such as offering subsidized loans to agricultural firms and small businesses, exempting social insurance payments and housing provident funds for corporate employers, deferring loan repayment deadlines for small- and medium-sized enterprises (SMEs), and instructing local companies to make full use of these policies. The government also stated that financial institutions should not blindly limit, rescind, or reduce loans SMEs. For enterprises severely impacted by the outbreak, their loans should be extended or renewed. Enterprises that encounter severe difficulties in production and operation as affected by the COVID-19 outbreak may apply for the deferred payment of social insurance premiums for six months. Eligible enterprises can directly enjoy value-added tax and corporate income tax preferences.
③ **Supply financing guarantee**. Specific measures include: playing the role of government-backed financing guarantee and providing credit enhancement to the agricultural industry. Government-backed financing guarantees and re-guarantee agencies halved guarantee fees for SMEs and strived to keep overall guarantee fees below 1% of the total loan amount. The central bank encouraged online financial companies such as "Ant Financial" to ease financing for small and micro-businesses.
④ **Relief special funds**. A Notice by the Ministry of Industry and Information Technology on February 9 2020, encouraged local government to set up special relief funds to increase support for SMEs that are severely impacted by the COVID-19 outbreak.

#### 3.3.3. Stabilizing agricultural production and products supply

Stabilizing agricultural production and products supply is an important means of guaranteeing people's livelihood. Two high-frequency words were associated with this topic: ensure supply (347) and stable production (178), accounting for 13.05% of the total frequencies amongst all topics.

Specific measures include: supporting the normal proceed of spring plowing season, stabilizing agricultural production in different areas, supporting farm mechanization in areas affected profoundly by the epidemic, taking measures to eliminate labor shortages, speeding up the restoration in the production and operation of livestock production and ensuring the supply of livestock and poultry products, ensuring smooth delivery of raw materials and products—such as the "green channel" policy for feed, breeding animals, meat, dairy products, seafood, supervising the implementation of the "vegetable basket" mayor's responsibility system to ensure the supply of "vegetable basket" products, presenting more support for the cold storage and preservation of agricultural products, enhancing the flexibility and risk-resistance of agricultural production and supply, granting market access to more countries and companies, shortening quarantine and review times, and opening green channel for agricultural products and food imports in key ports.

#### 3.3.4. Promoting agricultural products sale

There are four high-frequency words related to this topic: agricultural products sales (170), products allocation and transportation (130), green channel (119), and "point-to-point" sale (75), accounting for 12.28% of the total frequencies amongst all topics. This is manifested as follows:

① **Promote agricultural products sale**. After the outbreak of COVID-19, there is a vicious circle of "hard to buy and hard to sell" in agricultural products in many places, that is to say, the supply of agricultural products in cities has the problems of insufficient quantities and rising price. In contrast, the agricultural products of the corresponding producing area have difficulties in sales. Therefore, it is urgent to promote agricultural products sale. The specific measures include: using e-commerce platforms to directly supply agricultural products, enhancing the role of new-type agricultural operators such as cooperatives and family farms played in the sale of agricultural products, building a production and marketing docking platform to match information among agricultural enterprises to increase sales.
② **Supply green channel for transportation**. The specific measures include: Ensuring the transportation of agricultural products and agricultural production materials, opening a "green channel" for fresh agricultural products, such as vegetables, meat, egg, milk, aquatic products, grain crops, oils, to ensure smooth transportation, prohibiting setting up unauthorized roadblocks.

#### 3.3.5. Providing subsidies

For related industries that are affected by COVID-19 and suffer severe losses, it is urgent for the government to provide subsidies to help them tide over the difficulties. There are three related words: subsidy (246), allowance (170), and guaranteed purchase (48), accounting for 11.54% of the total frequencies amongst all topics.

This involves subsidies in many areas, including vegetable cultivation subsidies, poultry production subsidies, raw milk subsidies, pig production subsidies, insurance premium subsidies, subsidies for cold storage of agricultural products, etc. These subsidies have reduced the survival pressure and business risks of related enterprises. Meanwhile, a minimum purchase price for rice in 2020 has been promulgated, making it comparable to the minimum purchase price in 2019, giving farmers a stable expectation and thus increasing farmers' enthusiasm to grow grain. According to China's National Development and Reform Commission (NDRC), the cumulative subsidies issued across China reached $937.5 million during the COVID-19 period.

#### 3.3.6. Providing agricultural technology guidance and field management

Agricultural technology guidance and field management are essential guarantees for improving the supply capacity of agricultural products. There are four high-frequency words: agricultural technology (128), pest management (106), field management (66), and plant protection (49), accounting for 8.68% of the total frequencies amongst all topics. Specific measures can be reflected in the following four aspects:

① **Help with agricultural technology guidance**. Encouraging agricultural technicians to give technical guidance in the field; innovating agricultural technology training model, such as using online teaching platform, WeChat, QQ, short messaging service, agricultural technology promotion APPs, and other ways to carry out online agricultural technology guidance services. For example, Guangdong province has sent nearly 1,000 sci-tech experts to provide technical guidance to farmers to ensure the regular operation of spring plowing. Zhejiang province has provided sci-tech anti-epidemic manuals for farmers to speed up agricultural production.
② **Help with field management**. Poor field management will affect the yield and quality of agricultural production, so it is necessary to strengthen field management. Specific measures include: strengthening pests monitoring to reduce the damage; strengthening the integration of agricultural machinery and agronomy, and organizing large-scale agricultural machinery to carry out agricultural production and field management to alleviate the shortage of labor in field management.

#### 3.3.7. Providing assistance to poor farmers to reduce poverty

Assisting poor farmers is an important means to achieve the poverty alleviation goal of 2020 in China. There are two high-frequency words related to this topic: poverty alleviation (162) and assistance (75), accounting for 5.89% of the total frequencies amongst all topics.

Specific measures include: helping poor farmers return to their off-farm jobs; organizing poor farmers to carry out spring plowing preparations and helping them develop agricultural production; solving the problem of difficultly in selling agricultural and livestock products in poor areas, such as smoothing logistics and transportation in poor areas, organizing production and marketing docking, and using e-commerce platforms to sell products; increasing the investment of special funds for poverty alleviation; issuing microcredits for poverty alleviation; giving priority to supporting poverty alleviation projects; strengthening social security of poor farmers; providing direct and temporary assistance to poor farmers to ensure that all of them are guaranteed. For example, Huangmei County in Hubei Province expanded 4,000 public welfare jobs to help poor households out of the plight of the unemployable.

## 4. Policy implications

From the above analysis, we can find that the Chinese government has taken a series of emergency policies to ease the impacts of COVID-19, and these countermeasures are consistent with the impact aspects of COVID-19 on China's agricultural economy. However, although these measures have effectively guaranteed the stable development of agricultural production, there are still some shortcomings that need to be improved. Our suggestions for the future policies aimed at dealing the pandemic focus on the following four aspects:

First, more attention could be paid to compensation to farmers and agricultural enterprises due to their huge income loss resulting from COVID-19. As we elaborated in Section 3.3, most present measures aimed at resuming agricultural production and farmers' work—ranked number one. However, fewer actions were taken to recompense farmers' income loss, which is not conducive to stimulate the production enthusiasm of farmers and agricultural enterprises. Therefore, the following measures might be considered. ① Increasing subsidies to farmers. Providing subsidies for agricultural inputs to the severely affected areas to ensure the procurement of production materials. Increasing agricultural financial support funds to the recovery of production. Establishing a system for reporting, registering and compensating for agricultural production damage during the epidemic, and timely carrying out the compensation to the farmers to ensure the stability of agricultural production. ② Increasing policy-based financial support to agricultural enterprises. Agricultural enterprises play a critical role in helping small farmers to increase income in China [20]. Encouraging agricultural enterprises to provide production materials, technical services, and financial service support to small farmers and supporting them to purchase small farmers' products at a stable prize and volume. ③ Providing more public jobs in poor areas to farmers to increase their income. Public jobs can be developed in positions such as village cleanliness, road maintenance, forestry management and protection, and public facilities management and protection [21].

Second, more policies could be targeted at improving the efficiency of the agricultural supply chain system. Due to the short period of shortage and low adjustability in demand for agricultural products, the imperfect supply chain system of agricultural products is one of the main reasons for the severe impact of agriculture after the epidemic. Therefore, the following measures might be taken. ① Establishing an emergency agricultural stock mechanism to strengthen the smooth supply of key agricultural products. For example, storing an appropriate amount of meat and eggs to prepare for future meat shortfalls. Temporarily increasing the storage amount of corn to alleviate the supply difficultly of corn in Northeast China [22]. ② Building agricultural product processing systems and increasing support for agricultural product processing equipment. Encouraging agricultural enterprises to build agricultural product processing systems. Increasing support for agricultural product processing facilities in the process of preservation, drying, logistics, and storage, e.g., providing support to the operation of grain drying plants to increase the storage rate of rice and corn in Northeast China. Providing support to the operation of milk powder spraying plants to improve the sales of fresh milk.

Third, there is an urgent need to introduce measures to safeguard the import and export trade of agricultural products. It can be seen from Section 3.3 that there are few measures to address the risk of agricultural products trade from an international perspective, which contradicts with the complicated situation of the globalization of COVID-19. The following aspects might be paid attention. ① Strengthen international cooperation in the prevention and control of COVID-19. Strengthen trade consultations with major agricultural trading partners, avoiding them from using the epidemic as a means of technical trade barriers, adopting measures tailored to local conditions to carry out border trade, and ensuring that ports are no longer unilaterally closed. ② Guaranteeing the security of the global supply chain of key agricultural products, especially for the import of staple agricultural products such as grain, soybeans, oilseeds, pork, and cotton. The relevant import enterprises should be planned, arranged, and mobilized as early as possible to ensure the smooth operation of agricultural markets. China may consider reducing import tariffs on bulk agricultural products that have an essential impact on livestock and poultry farming and adopting more suitable measures for the import of fruits and vegetables from neighboring countries [23].

Lastly, it is necessary to ensure the effective implementation of policies. ① We can see that the Chinese government has promulgated many policies to deal with COVID-19; however, some policies are still at the document stage, need to be implemented timely. ② Many countermeasures are duplicated in various policies, such as the resuming of agricultural production has appeared in most policies listed in S1 Table, which will enhance the policy implementation cost. There is a need to form multi-sectoral cooperation in the implementation of policies to increase the implementation efficiency.

## 5. Conclusions

As COVID-19 continues to spread across the globe, it is essential to understand how China's agricultural economy is affected by COVID-19 and what emergency measures are government used to mitigating these impacts. Based on over 750,000 words collected from WeChat and Sina Weibo, this study employed web crawler technology and text mining method to empirically investigate the impacts of COVID-19 on China's agricultural economy and the Chinese government' measures to address these impacts.

The results show that: (1) the impact of COVID-19 on China's agricultural economy is mainly reflected in eight aspects. According to topic frequency, the order of these eight aspects followed the sequence of crop production (31.63%), agricultural products supply (27.93%), livestock production (17.98%), farmers' income and employment (8.41%), economic crop development (7.40%), agricultural products sales model (3.20%), leisure agriculture development (2.51%), and agricultural products trade (0.94%). (2) The government' countermeasures to deal with the impact of COVID-19 on agriculture are mainly reflected in seven aspects, which followed the sequence of resuming agricultural production and farmers' work (32.62%), providing financial support (15.94%), stabilizing agricultural production and products supply (13.05%), promoting agricultural products sale (12.28%), providing subsidies (11.54%), providing agricultural technology guidance and field management (8.68%), providing assistance to poor farmers to reduce poverty (5.89%).

Therefore, it can be seen that government's emergency countermeasures are basically consistent with the impact aspects of COVID-19 on China's agricultural economy. However, the order of government's countermeasures is not all in line with the order of impact aspects, which indicates that more-tailored policies should be implemented to minimize the negative impact of COVID-19 on China's agricultural economy. Our study also adds to the literature by empirically examined the impacts of COVID-19 on China's agricultural development and its countermeasures based on text mining techniques, which can provide more targeted implications for policymakers. A limitation of this study is that we only focus on the impacts from a relatively short time. Future research investigating the long-term impacts of COVID-19 on China's agricultural development should be conducted to provide more comprehensive suggestions for decision-makers. Another limitation is that we only examined the impacts of COVID-19 from a macro perspective and thus lacked an in-depth analysis from the micro perspective. Further research is required to conduct questionnaire-based investigations on individuals, such as farmers and agricultural enterprises, then using economic models to analyze the impacts of COVID-19 on their production, income, consumption, employment, and so on, thus providing a micro foundation for policy recommendations.

## Supporting information

S1 Table Representative policies by the Chinese government on each topic. (DOCX)
