# Supplementary material for: The influence of COVID-19 on agricultural economy and emergency mitigation measures in China: A text mining analysis
Source: PLoS One. 2020 Oct 23;15(10):e0241167. doi: 10.1371/journal.pone.0241167 (PMC7584181; doi:10.1371/journal.pone.0241167)
Supplement: S1 Table — (DOCX) [file pone.0241167.s001.docx]

**Table S1. Representative policies by the Chinese government on each topic.**

| Topic category | Enacted policies |
| --- | --- |
| Resuming agricultural production and farmers' work | ①On February 8, 2020, The State Council Responds to the COVID-19 Joint Defense and Joint Control Mechanism issued "*Notice on Earnestly Strengthening COVID-19 Scientific Prevention and Control and Orderly Achieving Enterprise Resumption of Work"*  ②February 9, 2020, Ministry of Industry and Information Technology issued *"Notice on Coping with the Work of COVID-19 Helping Small and Medium-sized Enterprises(SMEs) to Resume Production"*  ③February 15, 2020, General Office of the Ministry of Agriculture and Rural Affairs, General Office of the National Development and Reform Commission and General Office of the Ministry of Transport jointly issued *"Emergency Notice on Solving Current Practical Difficulties and Accelerating the Resumption of Production in the Breeding Industry"*  ④February 19, 2020, Ministry of Human Resources and Social Security, Ministry of Public Security, Ministry of Transport, National Health Commission and National Railway Group jointly issued *"Notice on Doing a Good Job of 'Point-to-Point' Service Guarantee for Returning to Work for Migrant Workers"*  ⑤February 27, 2020, General Office of the National Development and Reform Commission and General Office of the Ministry of Civil Affairs jointly issued *"Notice on Actively Playing the Role of Industry Associations and Chambers of Commerce to Support the Resumption of Private SMEs"*  ⑥March 2, 2020, General Office of the Ministry of Agriculture and Rural Affairs issued *"Notice on Coping with the Impact of COVID-19 to Expand Rural Labor Employment and Promote Farmers’ Income Increase"*  ⑦ March 2, 2020, Ministry of Agriculture and Rural Affairs, *"Notice on Further Optimizing Examination and Approval Services to Promote Agricultural Enterprises to Speed up Resumption of Production"*  ⑧March 26, 2020, Ministry of Agriculture and Rural Affairs and Ministry of Human Resources and Social Security jointly issued *"Implementation Plan for Expanding the Scale of Local Employment of Migrant Workers Returning to Their Hometowns"* |
| Providing financial support | ①January 31, 2020, Five departments including the People’s Bank of China and Ministry of Finance jointly issued *"Notice on Further Strengthening Financial Support and Prevention of COVID-19"*  ②February 1, 2020, Ministry of Finance issued *"Notice on Supporting the Work of Preventing and Controlling COVID-19 by Strengthening Financial Services"*  ③February 7, 2020, Ministry of Finance, The National Development and Reform Commission, Ministry of Industry and Information Technology, the People’s Bank of China and National Audit Office jointly issued *"Urgent Notice on Winning the COVID-19 Prevention and Control Battle, Strengthening the Key Support of Enterprise’s Financial Support for Epidemic Prevention and Control"*  ④February 7, 2020, Ministry of Finance and State Taxation Administration jointly issued *"Announcement on Supporting COVID-19 Prevention and Control Relevant Tax Policies"*  ⑤February 28, 2020, Ministry of Finance and State Taxation Administration jointly issued *"Announcement on Supporting the Value-Added Tax Policy for Individual Industrial and Commercial Households Resuming Work"* |
| Stabilizing agricultural production and products supply | ①January 30, 2020, Ministry of Agriculture and Rural Affairs, Ministry of Transport and Ministry of Public Security jointly issued *"Emergency Notice on Ensuring the Normal Circulation Order of ‘Vegetable Basket’ Products and Agricultural Production Materials"*  ②February 4, 2020, General Office of the Ministry of Agriculture and Rural Affairs issued *"Emergency Notice on Maintaining the Normal Order of Production and Marketing of Animal Husbandry and Guaranteeing the Supply of Meat, Egg and Milk Markets"*  ③February 12, 2020, The State Council Responds to the COVID-19 Joint Defense and Joint Control Mechanism issued *"Notice on Compacting the ‘Vegetable Basket’ Mayor Responsible for Making Work to Ensure Stable Production and Supply of Agricultural Products"*  ④February 14, 2020, General Office of the Ministry of Finance and General Office of the Ministry of Agriculture and Rural Affairs jointly issued *“Notice on Practically Supporting the Work of Stabilizing Production and Supply of Agricultural Products during the COVID-19 Prevention and Control period”*  ⑤February 17, 2020, 15 departments including The National Development and Reform Commission and Ministry of Industry and Information Technology jointly issued *"Notice on Doing Well the Production and Supply of Spring Plow Fertilizer in 2020 under COVID-19 Prevention and Control"*  ⑥February 19, 2020, Ministry of Agriculture and Rural Affairs issued *"Optimizing Multiple Measures for Administrative Examination and Approval to Ensure the Supply of Agricultural Materials and Agricultural Production"*  ⑦February 28, 2020, General Office of the Ministry of Agriculture and Rural Affairs issued *"Notice on Earnestly Supplying Agricultural Materials during the COVID-19 Prevention and Control Period"*  ⑧March 17, 2020, The National Development and Reform Commission and Ministry of Agriculture and Rural Affairs jointly issued *"Implementation Opinions on Supporting Private Enterprises to Develop Pig Production and Related Industries"*  ⑨March 19, 2020, Ministry of Agriculture and Rural Affairs, Ministry of Finance and China Banking and Insurance Regulatory Commission jointly issued *"Notice on Further Increasing Support and Promoting Stable Production and Supply of Live Pigs"*  ⑩March 19, 2020, Ministry of Agriculture issued *"Guidance on the Mechanized Production of Vegetables in Spring during the Outbreak"* |
| Promoting agricultural products sale | ①February 10, 2020, Ministry of Agriculture and Rural Affairs jointly issued *"Breakthrough Transportation Obstruction and Ensure Smooth Transportation of Agricultural Materials"*  ②February 11, 2020, Ministry of Agriculture and Rural Affairs issued *"Notice on Doing a Good Job in Selling Agricultural Products in Poor Areas during the COVID-19 Prevention and Control Period"*  ③February 14, 2020, General Office of the Ministry of Commerce issued *"Notice on Further Doing a Good Job in Matching the Production and Marketing of Agricultural Products during the COVID-19 Prevention and Control Period"*  ④February 26, 2020, Ministry of Transport and Ministry of Agriculture and Rural Affairs jointly issued *"Solve the ‘Difficulty of Entering the Village’ of Vehicles and Guarantee the Transportation of Agricultural Production Materials"* |
| Providing subsidies | ①February 28, 2020, The National Development and Reform Commission, Ministry of Finance, Ministry of Agriculture and Rural Affairs, National Food and Strategic Reserves Administration and Agricultural Development Bank of China jointly issued *"Notice on Announcement of the Minimum Purchase Price of Rice in 2020"*  ②April 2, 2020, Ministry of Finance issued *"Notice on the Government’s Full Use of the Financing Guarantee Function to Increase Credit for Small and Micro Enterprises and ‘Three Agriculture’ Subjects"* |
| Providing agricultural technology guidance and field management | ①March 2, 2020, The Central Leading Group for COVID-19 Work issued *"Notice on Issuing the Current Spring Cultivation Work Guidelines"* |
| Providing assistance to poor farmers to reduce poverty | ① February 17, 2020, The Poverty Alleviation Office of the State Council and Ministry of Finance jointly issued *"Notice on Actively Responding to the Impact of the COVID-19 and Strengthening the Financial Management of Special Poverty Alleviation Funds Projects to Ensure that the Goal and Tasks of Poverty Alleviation are Completed on Schedule"*  ②February 27, 2020, Ministry of Agriculture and Rural Affairs issued *"Actively Resolve the Impact of the Epidemic Situation and Fully Promote Industrial Poverty Alleviation"*  ③March 7, 2020, The Central Leading Group for COVID-19 Work issued *"Notice on Further Doing a Good Job in Safeguarding People in need during the Prevention and Control of Epidemic Situation"*  ④ March 13, 2020, Ministry of Agriculture and Rural Affairs issued *"Urgent Notice on Opening up Clogging and Promoting the Comprehensive Resumption of Production of Beekeeping Industry"* |
